# Activity‐Selectivity Trends in Electrochemical Urea Synthesis: Co‐Reduction of CO_2_ and Nitrates Over Single‐Site Catalysts

**DOI:** 10.1002/advs.202501882

**Published:** 2025-05-08

**Authors:** Qinglan Zhao, Yushen Liu, Yuan Zhang, Shangqian Zhu, Hongming Xu, Mohammad Farhadpour, Fei Xiao, Minghui Xing, Dapeng Cao, Xueping Qin, Tejs Vegge, Minhua Shao

**Affiliations:** ^1^ Department of Chemical and Biological Engineering The Hong Kong University of Science and Technology Clear Water Bay, Kowloon Hong Kong P. R. China; ^2^ School of Chemistry and Chemical Engineering Southeast University Jiangning District Nanjing Jiangsu 211189 P. R. China; ^3^ State Key Laboratory of Organic‐Inorganic Composites Beijing University of Chemical Technology Beijing 100029 P. R. China; ^4^ Department of Energy Conversion and Storage Technical University of Denmark Lyngby 2800 Kgs. Denmark; ^5^ Energy Institute, Chinese National Engineering Research Center for Control & Treatment of Heavy Metal Pollution, and CIAC‐HKUST Joint Laboratory for Hydrogen Energy The Hong Kong University of Science and Technology Kowloon Hong Kong P. R. China; ^6^ CIAC‐HKUST Joint Laboratory for Hydrogen Energy, Energy Institute The Hong Kong University of Science and Technology Clear Watery Bay, Kowloon Hong Kong 999077 P. R. China; ^7^ Guangzhou Key Laboratory of Electrochemical Energy Storage Technologies, Fok Ying Tung Research Institute The Hong Kong University of Science and Technology Guangzhou Guangdong 511458 P. R. China

**Keywords:** activity‐selectivity trend, C‐N coupling, co‐reduction reaction, electrocatalysis, single‐site catalyst

## Abstract

Electrochemical co‐reduction of carbon dioxide and nitrates (CO_2_NO_3_RR) holds promise for sustainable urea production. However, the sluggish kinetics of the sixteen‐electron transfer and unclear mechanistic understanding strongly impede its development. Here, combined experimental and computational approaches are employed to screen a series of metal phthalocyanine as model catalysts (MPcs, M = Zn, Co, Ni, Cu, and Fe) to uncover the activity‐selectivity trends in electrochemical CO_2_NO_3_RR. The theoretical simulations reveal that the thermodynamics of urea synthesis is significantly influenced by key intermediates, where the enhanced adsorption of *HOOCNO, coupled with reduced adsorptions of *N and *COOH, and moderate adsorption of *H_2_O, can significantly promote the urea production. ΔG_*HOOCNO_−ΔG_*N_−ΔG_*COOH_+ΔG_*H2O_ as a potential descriptor is proposed for predicting the efficiency of CO_2_NO_3_RR toward urea formation. The findings provide systematic guidance for the future design of high‐efficiency catalysts for urea electrosynthesis, addressing a crucial need for sustainable nitrogen fixation.

## Graphical abstract

In this work, a series of single‐site metal phthalocyanine catalysts (MPcs (M = Zn, Co, Ni, Cu, and Fe)) are screened to learn their activity‐selectivity relationship in the co‐reduction reaction of CO_2_ and nitrates toward urea formation, which can be significantly promoted by enhanced adsorption of *HOOCNO, reduced adsorptions of *N and *COOH, and moderate adsorption of *H_2_O.

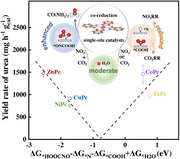

## 1 Introduction

Urea (CO(NH_2_)_2_) is a key reactant in the pharmaceutical industry and for producing fine chemicals. It also contains the highest nitrogen content of 46% in all solid nitrogenous fertilizers and is indispensable to modern agriculture, addressing the challenges posed by the world's growing population.^[^ ^1^, ^2^ ^]^ The current predominant industrial method for urea production was patented in 1922 with ammonia (NH_3_) and carbon dioxide (CO_2_) as reactants, which involves the Harber‐Bosch process to generate NH_3_ and further Bosch‐Meiser process to produce urea.^[^ ^3^ ^]^ Both processes require high‐temperature and high‐pressure synthetic conditions, consuming more than 2% of the global energy supply and generating more than 1% of the anthropogenic emission of greenhouse gases.^[^ ^4^ ^]^ Under the global ambition of reaching carbon neutrality by 2050, such energy‐intensive industrial manufacturing stimulates the emergence of sustainable methods for urea production.

The electrochemical co‐reduction reaction of CO_2_ and nitrates (CO_2_NO_3_RR) is a promising sustainable technology for urea production and a potential way to close both carbon and nitrogen cycles. The first work of urea electrosynthesis reported by Shibata et al. dates back to 1995.^[^ ^5^ ^]^ Since then, the progress remained relatively limited until 2020, when a report regarding the electrosynthesis of urea by Wang and colleagues reignited research enthusiasm in this field.^[^ ^6^ ^]^ Recently, many studies have been dedicated to improving the catalytic performance of electrocatalysts for urea production through the electrochemical co‐reduction of CO_2_ and nitrates/nitrites.^[^ ^7^, ^8^, ^9^, ^10^ ^]^ However, the electrochemical CO_2_NO_3_RR for urea production is still in its infancy.^[^ ^11^ ^]^ The development of active and selective C−N bond‐forming electrocatalysts for electrosynthesis of organonitrogen compounds, in general, constitutes a substantial materials design challenge and underpins the need for an in‐depth understanding of,^[^ ^12^ ^]^ e.g., the complex 16‐electron transfer co‐reduction reaction mechanism involving C−N bond formation, which limits the design and development of highly efficient catalysts.

Great efforts have been dedicated to unraveling the mechanisms of this co‐reduction reaction system. The C−N coupling toward practical urea production has been reported on various electrocatalysts through bonding between different C‐species (CO_2_, *CO_2_, *CO, and *COOH) and N‐species (*NO_2_, *NO, *H_2_NOH, *NH_2_, *NH, and *N).^[^ ^7^, ^8^, ^9^, ^10^, ^13^, ^14^, ^15^, ^16^, ^17^, ^18^, ^19^, ^20^, ^21^, ^22^, ^23^, ^24^ ^]^ In some theoretical studies, it was reported that *CO and *NH are key intermediates on Cu (100) surfaces for C‐N coupling toward urea formation at low overpotentials.^[^ ^25^ ^]^ In addition, different metal (100) surfaces were selected as model facets to study the C−N formation mechanism.^[^ ^26^ ^]^ It was found that the adsorption energies of hydrogen and oxygen atoms are correlated with the catalytic performance. While insights from previous work can potentially benefit catalyst design for urea electrosynthesis, these studies either focus on specific catalysts to explain experimental results or rely solely on theoretical simulations using data from early reports. A combination of experimental work and theoretical calculations is highly desirable and necessary to deepen our mechanistic understanding of C−N coupling in the electrochemical co‐reduction system for urea formation.^[^ ^11^, ^27^, ^28^, ^29^ ^]^

In the present work, a series of metal phthalocyanines (MPcs) with active M (metal)−N (nitrogen) single sites are employed as model catalysts. The performance of M−N sites for C−N coupling in urea formation, as well as their performance in competitive reactions, including carbon dioxide reduction reaction (CO_2_RR), nitrate reduction reaction (NO_3_RR), and hydrogen evolution reaction (HER), were investigated and discussed. A well‐established activity‐selectivity landscape of CO_2_NO_3_RR for urea synthesis over this series of MPc model catalysts with well‐defined M−N_4_ moieties opens the door to a consistent and reliable interpretation, combining both experimental and theoretical efforts. This approach allows for the exploration of the relationship between fundamental properties and electrochemical performance.

## 2 Results and Discussion

### 2.1 Electrocatalytic Urea Production on MPcs

**Figure 1:**
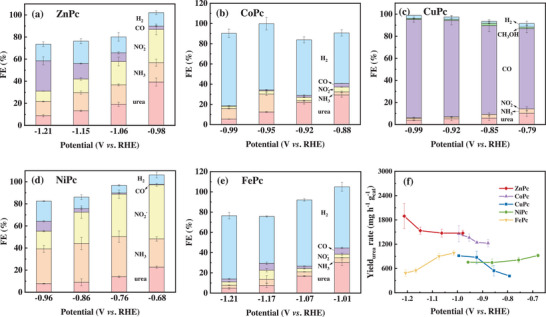
FEs of various products produced on MPc catalysts under different potentials: a) ZnPc, b) CoPc, c) CuPc, d) NiPc, e) FePc; and f) comparison of yield rates of urea on the MPc catalysts at different potentials.

The single‐site metal phthalocyanine catalysts offer the platform for mechanistic studies as a structural model of M−N_4_ units to elucidate how active metal centers affect the catalytic activity and selectivity converting CO_2_ and NO_3_^−^ toward urea formation. As the MPc catalysts exhibit different electrocatalytic activities, their performance in the co‐reduction reaction was evaluated using the H‐type cell in various potential windows (**Figure** **1**a–e). The potential of −1.0 V_RHE_ (V versus the reversible hydrogen electrode, RHE) was selected to compare these MPc catalysts, as it represents an overlapping point for all tested catalysts with obvious differences in electrochemical performance. ZnPc reached the highest Faradaic Efficiency of urea (FE_urea_) and the highest yield rate at the potential close to −1.0 V_RHE_, indicating the highest selectivity and activity among the tested MPc catalysts. CoPc achieved approximately the same yield rate but a relatively low FE_urea_, while FePc delivered the second‐highest FE_urea_ but a relatively low yield rate. The two remaining catalysts, CuPc and NiPc, exhibited relatively low FE_urea_ and yield rates. In detail, the FE_urea_ corresponding to selectivity was ranked in the order of ZnPc (39.30±3.65% at −0.98 V_RHE_) > FePc (30.15±2.84% at −1.01 V_RHE_) > NiPc (7.79±0.34% at −0.96 V_RHE_) > CoPc (5.40±0.18% at −0.99 V_RHE_) > CuPc (3.96±0.35% at −0.99 V_RHE_). Correspondingly, the yield rate reflecting electrocatalytic activity and selectivity was in the following order of ZnPc (1474±70 mg h^−1^ g_cat_^−1^) > CoPc (1458±200 mg h^−1^ g_cat_^−1^)> FePc (982±63 mg h^−1^ g_cat_^−1^) > CuPc (915±24 mg h^−1^ g_cat_^−1^) > NiPc (754±8 mg h^−1^ g_cat_^−1^) as shown in Figure 1f. The total FEs are not exactly equal to 100%, of which the missing part may be due to the residue liquid products from CO_2_RR such as HCOOH, methanol, and ethanol.^[^ ^24^, ^30^ ^]^

### 2.2 Identification of the Stable M−N_4_ Units

Before further exploring mechanistic insights behind the selectivity and activity orders, conducting in situ characterizations on the MPc catalysts is crucial since the metal centers of these molecular catalysts can aggregate during electrochemical measurements.^[^ ^31^, ^32^, ^33^ ^]^ In situ Raman has been proven as an effective technique to monitor the dynamic changes of CoPc catalyst during CO_2_RR.^[^ ^31^ ^]^ In this work, in situ Raman experiments were carried out to confirm the maintained M−N_4_ structure of MPc catalysts at the selected potential. The potential window of interest for each catalyst in electrochemical measurements was tested by in situ Raman experiments. As shown in Figure S1 (Supporting Information), the MPc catalysts show their characteristic bands in the Raman spectra compared to the bare glassy carbon electrode (GCE). No new band or band shift was observed in the potential window of interest for each catalyst (from the open circuit potential (OCP) to −1.29V_RHE_ for ZnPc; from OCP to −1.09V_RHE_ for NiPc, CoPc, and CuPc) except FePc. However, the in situ Raman spectra of FePc only displayed new bands at 1069, 1127, and 1535 cm^−1^, a strengthened band at 1300 cm^−1^, and a weakened band at 1403 cm^−1^ until the potential going below −1.19 V_RHE_. Therefore, all MPc catalysts maintained their original frameworks at the selected potential of −1.0 V_RHE_, demonstrating the suitability of M−N_4_ structural units for further theoretical exploration of the CO_2_NO_3_RR mechanism on the MPc catalysts.

Our experimental results show a good agreement with the previous report that CoPc exhibited aggregated Co metal centers for CO_2_RR until the potential of −1.0 V_SHE_ (V versus the standard hydrogen electrode, SHE) or more negative.^[^ ^31^ ^]^ Similarly, these MPc catalysts were claimed to display ignorable structural changes from 0.2 to −1.0 V_RHE_ during CO_2_RR using in situ X‐ray absorption near‐edge structure (XANES) spectra.^[^ ^34^ ^]^ Another work showed that the CuPc structure started to change at −0.66 V_RHE_, partially reducing the Cu(II) to Cu(I).^[^ ^32^ ^]^ The timing of metal aggregate formation in MPc active materials during reactions may vary across different reaction systems, influenced by the specific local environment. In addition, it has been reported that the composition of catalysts also affects metal aggregate formation. For example, simply increasing the conductivity additives can prevent excessive agglomeration of Cu clusters in CuPc catalysts during reactions.^[^ ^33^ ^]^

These MPc samples show stable yield rates of urea for continuous five cycling tests, as shown in Figures S2–S6 (Supporting Information). We also measured the concentrations of metal ions in electrolytes after catalytic reactions. As shown in Table S1 (Supporting Information), all concentrations of metal ions are no more than 1.02 ppb, ruling out the possibility of dissolution of catalysts during CO_2_NO_3_RR. In addition, the scanning electron microscope (SEM) images of these MPc samples after catalytic reactions show ignorable changes (Figure S7, Supporting Information), further indicating the stability of the samples.

### 2.3 Mechanistic Understanding of Urea Formation on MPcs

**Figure 2:**
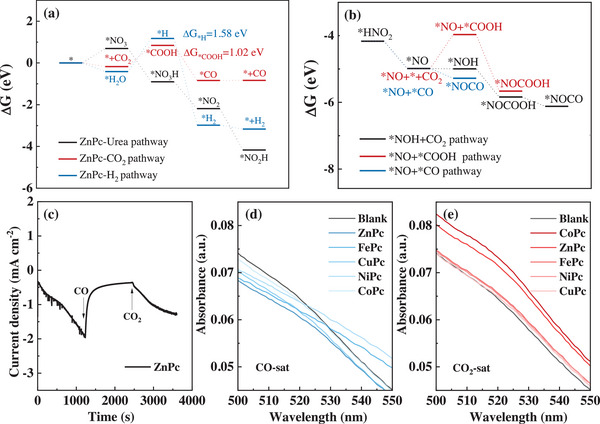
a) Simulated pathways toward formation of urea, CO_2_, and H_2_; b) the diverted pathways for the first C−N couplings and the selected configuration of *NOCOOH; c) The current density versus time curves of different MPc catalysts at an applied potential of −1.0 V_RHE_ without iR compensation with CO and CO_2_ shifting on ZnPc catalyst; d, e) UV‐vis spectra of electrolytes after 1 h electrocatalytic reaction on MPc catalysts with injected gas using (d) CO or (e) CO_2_. (Calculations were conducted under pH = 6.8 at the potential of −1.0 V_RHE_).

Density functional theory (DFT) calculations were performed to understand the catalytic trends of C−N coupling toward urea production on the above MPc catalysts with M−N_4_ units. We started with the investigation of possible pathways on the ZnPc catalyst, which showed the highest selectivity and yield rate. In this complex co‐reduction system with co‐existence of many reagents including CO_2_, NO_3_^−^, and H_2_O, the NO_3_^−^ reduction is more favorable than the competitive CO_2_RR and HER due to the lower Gibbs free energy barrier of *NO_3_ adsorption (0.69 eV, Bader charge transfer of 0.70 e net charge as shown in Figure S8, Supporting Information) than that of *COOH (1.02 eV) and *H (1.58 eV) adsorption in electrochemical reaction process as shown in **Figure** **2**a. This is also in accordance with the experimental results that the N‐containing products (urea, NH_3_, and NO_2_^−^) were produced more than the formed CO and H_2_ at the potential of −0.98 V_RHE_ (Figure 1a). In addition, more NO_3_RR products were also obtained on ZnPc when compared with the HER in the Ar‐saturated control experiments at the applied potential of −0.97 V_RHE_ (Figures S9a and S10, Supporting Information), which can be explained by the insufficient *H produced by the HER process with a higher Gibbs free energy barrier in the electrochemical reaction process. However, HER occurs predominantly in the independent CO_2_RR system (Figure S9b, Supporting Information), which may be due to the stronger adsorption of H_2_O with lower adsorption energy (−0.41 eV) than CO_2_ (−0.18 eV) on ZnPc surface (Table S2, Figures S11 and S12, Supporting Information). Therefore, the theoretical analysis also matches the experimental data. Then, we continued the pathway of *NO_3_ reduction on ZnPc based on the theoretically favorable intermediates with the lowest energy states until the diversion toward NH_3_ through the subsequent protonation or urea formation through the C−N coupling at the intermediate of *NOH.

**Figure 3:**
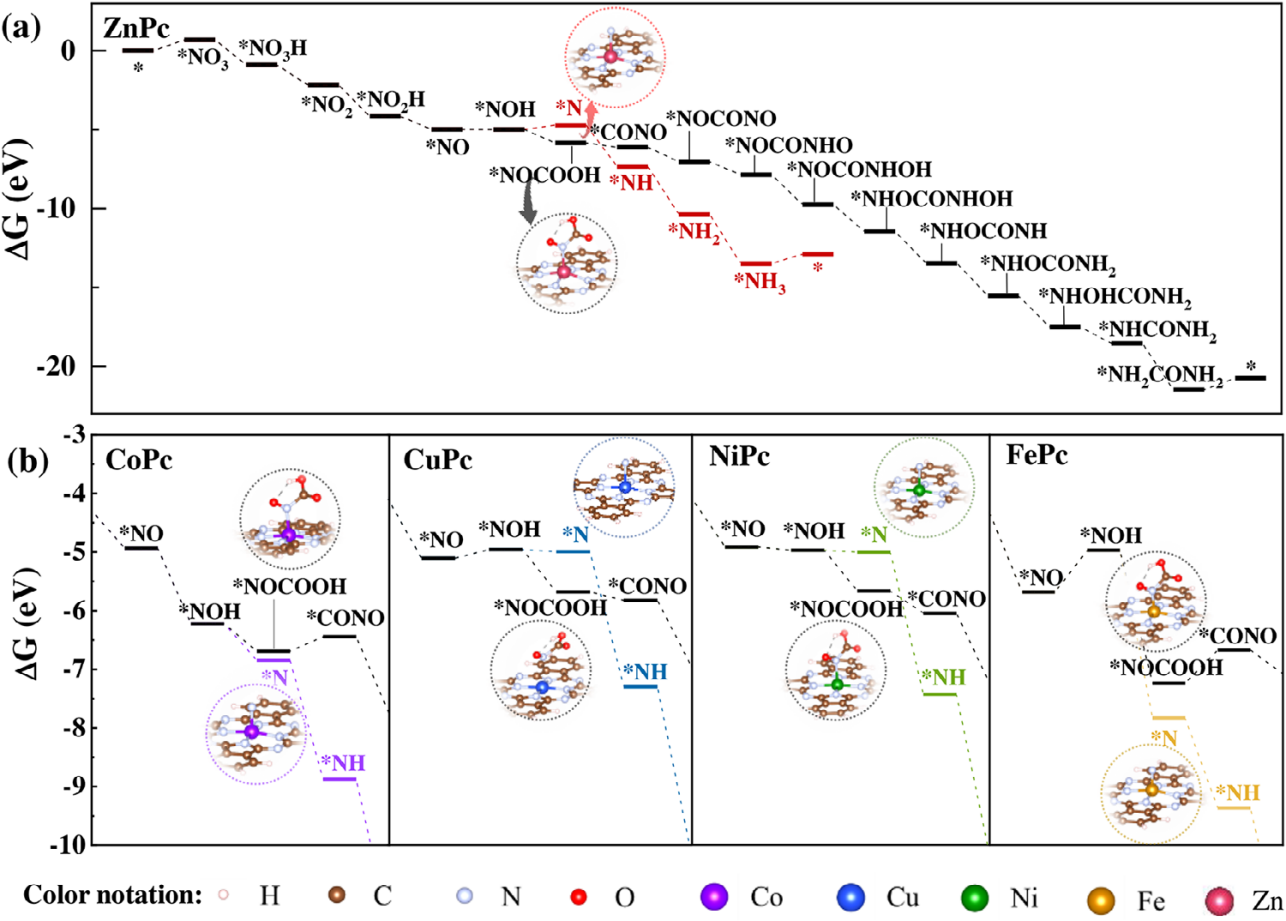
a) Pathway of CO_2_NO_3_RR toward urea formation and the one of NO_3_RR toward NH_3_ formation on ZnPc catalyst; b) the diversions toward *N or *NOCOOH steps in the NH_3_ pathway or urea pathway for CoPc, CuPc, NiPc, and FePc catalysts (inserts showing the adsorption configurations of *N and *NOCOOH on each catalyst).

Generally, there are three main types of C‐species for C−N coupling, including *CO, *COOH, and CO_2_ (*CO_2_).^[^ ^11^ ^]^ As shown in Figure 2b, the pathway through *CO showed the lowest downhill energy trend, suggesting that it is a very promising intermediate for C−N coupling. To check this possibility, we conducted the control experiment by shifting from CO_2_ to CO into the co‐reduction system. The current density suddenly decreased when the injected gas was shifted to CO (Figure 2c; Figure S13, Supporting Information), which was applied to all MPc catalysts. In addition, no urea product was detected when injecting CO into the system, with no absorbance of urea at 518 nm, as shown in Figure 2d. Thought it could be due to low solubility of CO, *CO and CO should co‐exist and become interconvertible at an equilibrium state when CO was introduced,^[^ ^35^, ^36^ ^]^ In this case, we excluded *CO as the intermediate for C−N coupling here. Then, the possible pathways should be through the coupling with CO_2_ or *COOH, which can be formed when CO_2_ was introduced into the system with an absorbance of urea shown at 518 nm (Figure 2e). To identify CO_2_ or *COOH for the C−N coupling, we calculated the adsorption energies for their pathways on ZnPc (Figure 2b). The formation of *COOH faced a much higher energy barrier compared to CO_2_, making it an unfavorable intermediate for C−N coupling. Therefore, the incoming gas CO_2_ was selected for the first C−N coupling step with *NOH, as shown in **Figure** **3**. The second C−N coupling soon occurred between *CONO and *NO, followed by subsequent protonation based on the Eley‐Rideal mechanism to complete the pathway toward urea formation. As NO_3_RR toward NH_3_ formation is also another important competitive reaction in this system, it was also discussed and compared, as shown in Figure 3. The pathway toward urea production shares part of the NH_3_ formation pathway with the diversion at the intermediate of *NOH (configurations shown in Figure S14, Supporting Information), continuing the protonation forming *N or not. The subsequent downhill step of forming *NOCOOH (configurations shown in Figure S15, Supporting Information) in the urea pathway (ΔG = −0.84 eV) compared with the uphill step of *N formation in the NH_3_ pathway (ΔG = 0.28 eV) favors the urea production in this co‐reduction system, which also matches the experimental results (Figure S9a, Supporting Information).

Subsequently, the urea formation pathways on other MPc models (M = Cu, Co, Fe, and Ni) started from the adsorption of *NO_3_ (configurations shown in Figure S10, Supporting Information) were investigated using DFT method. As shown in **Figure** **3**a; Figures S16–S19, and Table S3 (Supporting Information), all Gibbs free energy change diagrams demonstrated a similar tendency to that of ZnPc (Figure 3a). The only exception exists in steps of FePc and CoPc (Figure 3b). At the potential of −1.0 V_RHE_, the urea synthesis pathway for FePc and CoPc does not continuously decline. Instead, both catalysts display energy barriers during the *CONO formation step (Figure 3b), with 0.25 eV for CoPc and 0.57 eV for FePc, respectively. Notably, *NOH is the shared intermediate diverging both urea formation reaction and NO_3_RR pathways (Figure 3a and b). FePc and CoPc presented a higher energy state for *NOH than *N. Significantly, the FePc showed its highest energy barrier at the *NOH formation step, which is higher than the *N formation step. It makes NO_3_RR more theoretically favorable on CoPc and FePc catalysts that fit the increasing produced ratio of NH_3_ to urea with decreasing applied potentials. In addition, the CO_2_RR shows a downhill trend in the Gibbs free energy change, but experimental results showed their poor catalytic CO_2_RR performance (Figures S20b, S23b, and S24, Supporting Information). This finding indicates that the intermediates of CO_2_RR, like *COOH or *CO, may not participate in the C−N coupling process, which is also consistent with the discussions above.

### 2.4 Descriptors of C−N Coupling toward Urea Formation

**Figure 4:**
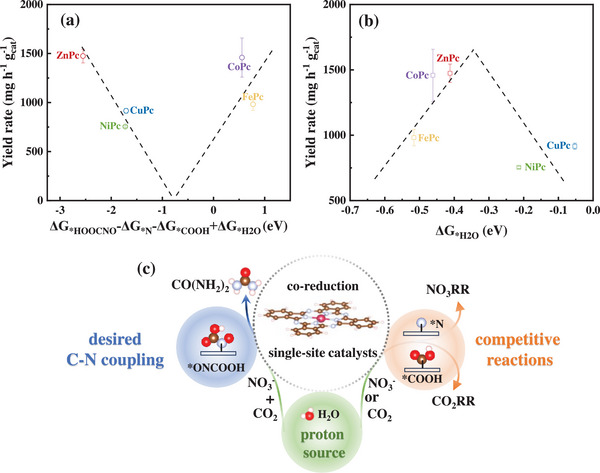
a) The trend of the experimental yield rates (y‐axis) and the selective DFT calculated electrochemical steps as the formulated function descriptors (x‐axis) for urea formation on various MPc catalysts in the CO_2_NO_3_RR system; b) volcano‐shaped correlation of the experimental yield rates (y‐axis) and the selective DFT calculated chemical step of *H_2_O adsorption (x‐axis); and c) scheme of illustrating the roles of adsorbed intermediates in the above formulated function descriptors.

Based on the above discussion, we believe that catalytic activity and selectivity of the catalysts toward urea production are mainly determined by the binding properties of key electrochemical intermediates *N, *COOH, *HOOCNO, and *H_2_O. Specifically, the adsorption of *HOOCNO is crucial, and it is the first C−N coupling step toward urea formation. The adsorption of *N is the step determining the diversion from the urea pathway to the competitive NO_3_RR. The adsorption of *COOH is the rate‐determining step in the competitive CO_2_RR. The adsorption of *H_2_O participates in the entire pathway of urea formation being the proton source. Therefore, the calculated adsorption energy (Table S4, Supporting Information) of *HOOCNO (ΔG_*HOOCNO_), *N (ΔG_*N_), *COOH (ΔG_*COOH_), and *H_2_O (ΔG_*H2O_) were used to plot the formulated function as descriptors toward urea formation with the yield rates representing activity and selectivity on these MPc catalysts. It should be noted that, in our computations, the thermodynamics of intermediates is considered a reliable predictor of catalytic performance due to the linear Bronsted–Evans–Polanyi relationship between thermodynamics and kinetics, and the effects of solvents and ions are not included, which are beyond the scope of current study. As demonstrated in **Figure** **4**a, the CO_2_NO_3_RR activity toward urea production follows an inverse volcano plot with the function of ΔG_*HOOCNO_−ΔG_*N_−ΔG_*COOH_+ΔG_*H2O_ on various transition metal‐based MPc catalysts. In this plot, the further the descriptor value of a catalyst is from the vertex, the better its performance in producing urea may be. Considering the close correlation between the adsorption of intermediates and the electronic structures, regulation of various transition metals with different electronic states can fundamentally adjust the adsorption of the above key intermediates to facilitate urea production in the CO_2_NO_3_RR system.

It is noted that the moderate adsorption of the chemical step adsorbing *H_2_O (ΔG_*H2O_) is important. The adsorption of *H_2_O (configurations shown in Figure S11, Supporting Information) not only determines the competitive HER but also affects the proton‐coupled electron transfer in the electrochemical steps of the urea pathway and competitive NO_3_RR. The adsorption of *H_2_O (ΔG_*H2O_) was thus plotted with the yield rate of urea on various MPc catalysts, as shown in Figure 4b. The CO_2_NO_3_RR activity toward urea production demonstrated a typical volcano‐trend relationship with the adsorption of *H_2_O (ΔG_*H2O_) on various transition metal‐based MPc catalysts. Among them, the ΔG_*H2O_ of ZnPc and CoPc are close to the optimal value, which is neither too strong nor too weak. Therefore, the promoted urea production performance of ZnPc and CoPc can be due to the enhanced adsorption of *HOOCNO, reduced adsorption of *N and *COOH, and moderate adsorption of *H_2_O (Figure 4c). The charge transfer is further analyzed for the key intermediate involved in the rate determining step, i.e., *NO on ZnPc, FePc, CuPc, and NiPc, and *NOCOOH on CoPc, with the corresponding charge density difference distribution shown in Figure S25 (Supporting Information). However, the amount of transferred charge is too small to make a difference, which further indicates the performance of urea production was not determined by a single step but affected by complex multiple steps.

## 3 Conclusion 

In summary, the co‐reduction reaction of CO_2_ and nitrate was studied on a series of metal phthalocyanines (MPcs, M = Zn, Co, Ni, Cu, and Fe), which acted as model catalysts offering a way to unambiguously investigate the role of the isolated transition metal center with identical local coordination environment on the activity and selectivity toward urea formation, using a combination of electrochemical evaluations, in situ Raman analyses, and DFT calculations. The high performance of urea production is not only determined by the key electrochemical step of *HOOCNO adsorption but also affected by the chemical step of *H_2_O adsorption. In addition, the electrochemical steps of adsorbing *N and *COOH intermediates in the competitive nitrate reduction reaction and CO_2_ reduction reaction also affect the performance of urea production. As a result, the volcano correlation between the DFT‐calculated Gibbs free energy difference of ΔG_*HOOCNO_−ΔG_*N_−ΔG_*COOH_+ΔG_*H2O_ and experimentally obtained yield rates of urea is observed, suggesting that ΔG_*HOOCNO_−ΔG_*N_−ΔG_*COOH_+ΔG_*H2O_ can be used as a potential descriptor of the CO_2_NO_3_RR toward urea production. It is further confirmed by DFT calculations that the moderate adsorption of *H_2_O, being neither too strong nor too weak, promotes urea production in the co‐reduction system. These mechanistic insights elucidated by the isolated metal‐centered model catalysts can facilitate the rational design of high‐efficiency catalysts for the CO_2_ and nitrate co‐reduction reaction and guide element screening in the future.

## 4 Experimental Methods

### Electrochemical Measurements

The electrochemical performances of the metal phthalocyanine catalysts were evaluated in an H‐type cell using a CHI workstation. The catalyst ink was prepared by mixing 3.5 mg of commercial metal phthalocyanines and 1.5 mg of carbon black with 1950 µL of isopropanol and 50 µL of 5 wt.% Nafion solution, followed by intensive ultrasonication. 10 µL of the well‐dispersed ink was drop‐cast onto a glassy carbon electrode (GCE) with an electrode area of 0.785 cm^2^ to form a uniform catalyst layer. Calibrated Ag/AgCl electrode and Pt mesh with an area of 2×2 cm^2^ were used as the reference and the counter electrode, respectively. Nafion 117 membrane was used as a separator. 30 or 40 mL (depending on the volume of reactor) of 0.1 mol L^−1^ KHCO_3_/0.01 mol L^−1^ KNO_3_ solution and 30 or 40 mL of 0.5 mol L^−1^ KHCO_3_ electrolyte were used as catholyte and anolyte, respectively. Before the electrochemical experiments, CO_2_ (99.999%, Asia Pacific Gas Enterprise Co., LTD) was bubbled into the catholyte for 1 h at a flow rate of 30 sccm, with continuous purging and stirring at a rate of 800–1000 rpm during experiments. For stability tests, each cycle run for 1 h followed by replacement of fresh electrolyte. All potentials were converted to the reference scale of reversible hydrogen electrode (RHE) according to the following equation:

(1) $$E\left(vs.\;\mathrm{RHE}\right)=E\left(vs.\;\mathrm{Ag}/\mathrm{AgCl}\right)+E_{\mathrm{Ag}/\mathrm{AgCl}}+0.0591\times \left(6.8-1.1\right)$$

where *E*_Ag/AgCl_ (against RHE) was the potential of the Ag/AgCl reference electrode measured in H_2_‐saturated 0.1 M HClO_4_ solution with pH = 1.1. If not particularly mentioned, *iR* compensation was conducted manually after the measurements.

### Product Analyses

The gas products were analyzed using an online GC2060 gas chromatography machine, with sampling conducted at the 15^th^, 28^th^, 41^st^, and 54^th^ min, sequentially. The current density was calculated according to the total charges collected in the 30 s prior to each sampling point. The liquid products including urea, ammonia, and nitrite were measured by the ultraviolet‐visible spectroscopy (UV‐vis) method. Three independent measurements were used to test each catalyst to ensure the accuracy of the experiments.

The diacetylmonoxime method was used to measure the concentration of produced urea.^[^ ^24^, ^37^ ^]^ 100 mL concentrated phosphoric acid, 300 mL concentrated sulfuric acid, 600 mL deionized water, and 100 mg ferric chloride were mixed to prepare acid‐ferric solution. 5 g DAMO and 100 mg TSC were dissolved in 1000 ml deionized water to prepare diacetylmonoxime (DAMO)‐thiosemicarbazide (TSC) solution. Then, 1 ml sample electrolyte, 2 mL acid‐ferric solution, and 1 mL DAMO‐TSC solution were mixed, followed by 30 min boiling in a water bath. After cooling down to room temperature, the absorbance of the above mixture solution was obtained at 518 nm on a Cary 5000 UV‐Vis‐NIR spectrophotometer. The calibration curve of concentration‐absorbance was generated using the absorbance of standard urea solutions, cited from this previous work (Figure S26, Supporting Information).^[^ ^24^ ^]^

The indophenol blue method was used to measure the concentration of produced ammonia.^[^ ^24^, ^38^ ^]^ 2 ml sample electrolyte was mixed with 2 ml of 1 mol L^−1^ NaOH solution containing 5wt% salicylic acid, 5wt% sodium citrate, 1 ml of 0.05 mol L^−1^ NaClO, and 0.2 ml of 1wt% sodium nitroferricyanide. After 2 h at room temperature, the absorption spectrum was acquired on a Cary 5000 UV‐Vis‐NIR spectrophotometer with the absorbance obtained at 657 nm. The calibration curve of concentration‐absorbance was generated with the absorbance of standard ammonia solutions, cited from this previous work (Figure S27, Supporting Information).^[^ ^24^ ^]^

A modified N‐(‐1‐naphthyl) ethylenediamine dihydrochloride spectrophotometric method was used to measure the concentration of produced nitrite.^[^ ^24^, ^39^ ^]^ 2 ml of the sample electrolyte was mixed with 0.1 mL of 4‐aminobenzenesulfonamide aqueous solution (10 g L^−1^ in 10 wt% HCl). After the reaction for 8 min, 0.1 mL of *N‐*(‐1‐naphthyl) ethylenediamine dihydrochloride aqueous solution (1 g L^−1^) was added into the above mixture, followed by further reaction for 10 min in the dark. The UV‐vis absorption spectrum was acquired at 540 nm on a Cary 5000 UV‐Vis‐NIR spectrophotometer. The calibration curve of concentration‐absorbance was generated with the absorbance of standard NaNO_2_ solutions, cited from this previous work (Figure S28, Supporting Information).^[^ ^24^ ^]^

### Characterizations

Inductively coupled plasma mass spectrometry (ICP‐MS) measurements were conducted on an Agilent 7700X. Field emission scanning electron microscopy (FESEM) images were captured on a FESEM microscope (JSM 6700F). In situ Raman experiments were performed using a customized flow cell on a Renishaw InVia Raman microscope with a 532 nm laser and a grating of 1800 grooves/mm. The in situ Raman spectra were collected from 200 to 2500 cm^−1^ with 10‐second exposure time. A Gamry Interface 5000 instrument was used to conduct the parallel controlled potential electrochemical tests. Each electrochemical test was performed at a certain potential for 480 seconds in a CO_2_‐saturated electrolyte of 0.1 mol L^−1^ KHCO_3_/0.01 mol L^−1^ KNO_3_. The spectrum of bare GCE in the same electrolyte was also acquired for comparison.

### Computational Methods

All spin‐polarized density functional theory (DFT) calculations were performed by the Vienna Ab initio Simulation Package (VASP)^[^ ^40^ ^]^ based on the projector‐augmented wave (PAW) method.^[^ ^41^ ^]^ The Perdew‐Burke‐Ernzerhof (PBE) functional was used to estimate the exchange‐correlation energies.^[^ ^42^ ^]^ The plane‐wave‐basis set energy cutoff was 400 eV. The convergence criteria were 10^−5^ eV for energy and 0.02 eV Å^−1^ for force. The partial occupancies were set by Gaussian smearing with a width of 0.05 eV. Dipole corrections were introduced along the z direction. A single metal phthalocyanines molecule was placed in a 25 Å × 25 Å × 20 Å unit cell as the simulation model. The van der Waals correction was done by introducing the DFT‐D3 method of Grimme with zero‐damping function. The Gibbs’ energy change of the CO_2_RR, NO_3_RR, HER, and urea formation processes were calculated with the following equation based on the computational hydrogen electrode (CHE) model:^[^ ^43^ ^]^

(2) $$\Delta G=\Delta E_{DFT}+\Delta E_{ZPE}-T\Delta S+pH\times \mathrm{kTln}10-eU$$

where Δ*E_DFT_* was the total energy of a certain optimized structure with reaction intermedia from the DFT simulation. Δ*E_ZPE_* was the zero‐point energy calculated from vibrational frequencies, *T* was the temperature (298.15 K), *S* was the entropy obtained from standard thermodynamics tables^[^ ^44^ ^]^ or from vibrational frequencies, *pH* was the pH value of electrolyte in reaction condition, k was the Boltzmann constant, and *U* was the applied electrode potential versus revisable hydrogen electrode (RHE). In this work, Δ*E_ZPE_* and *S* were directly obtained from frequency calculations using VASPKIT.^[^ ^45^ ^]^ Bader charge analysis was used to explore the electron transfer between NO_3_^−^ and catalyst substrates.^[^ ^46^, ^47^ ^]^

## Conflict of Interest

The authors declare no conflict of interest.

## Supporting information



Supporting Information

## Data Availability

The data that support the findings of this study are available from the corresponding author upon reasonable request.
