# Supplementary material for: Activity‐Selectivity Trends in Electrochemical Urea Synthesis: Co‐Reduction of CO2 and Nitrates Over Single‐Site Catalysts
Source: Adv Sci (Weinh). 2025 May 8;12(27):2501882. doi: 10.1002/advs.202501882 (PMC12279245; doi:10.1002/advs.202501882)
Supplement: Supplementary file 1 — Supporting Information [file ADVS-12-2501882-s001.pdf]

ADVANCED SCIENCE
Open Access

Supporting Information

for *Adv. Sci.*, DOI 10.1002/advs.202501882

Activity-Selectivity Trends in Electrochemical Urea Synthesis: Co-Reduction of CO_2 and Nitrates Over Single-Site Catalysts

Qinglan Zhao, Yushen Liu, Yuan Zhang, Shangqian Zhu, Hongming Xu, Mohammad Farhadpour, Fei Xiao, Minghui Xing, Dapeng Cao, Xueping Qin, Tejs Vegge and Minhua Shao**

Supporting Information

Activity-Selectivity Trends in Electrochemical Urea Synthesis: Co-reduction of CO_2 and Nitrates over Single-Site Catalysts

Qinglan Zhao, Yushen Liu, Yuan Zhang, Shangqian Zhu, Hongming Xu, Mohammad Farhadpour, Fei Xiao, Minghui Xing, Dapeng Cao, Xueping Qin, Tejs Vegge, and Minhua Shao**

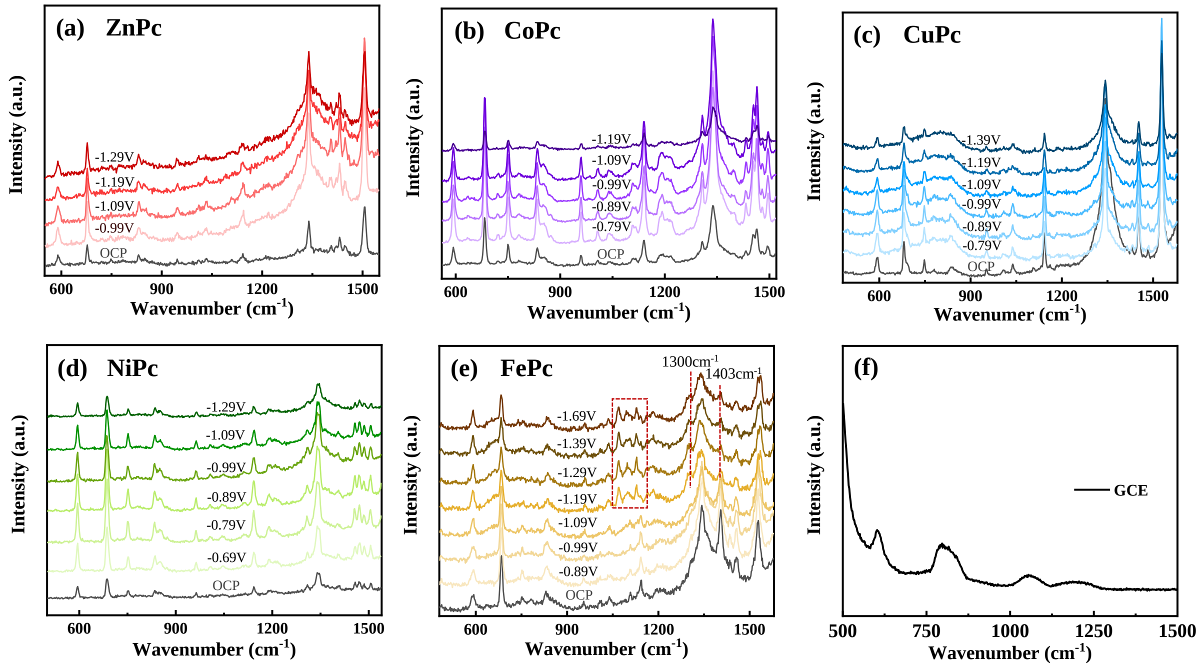

Figure S1. *In situ* Raman spectra of (a) ZnPc, (b) CoPc, (c) CuPc, (d) NiPc, and (e) FePc during the CO_2NO_3RR at different potentials (without iR correction); (f) Raman spectrum of bare GCE acquired in the electrolyte of CO_2-saturated 0.1 M $KHCO_3$/0.01 M KNO_3.

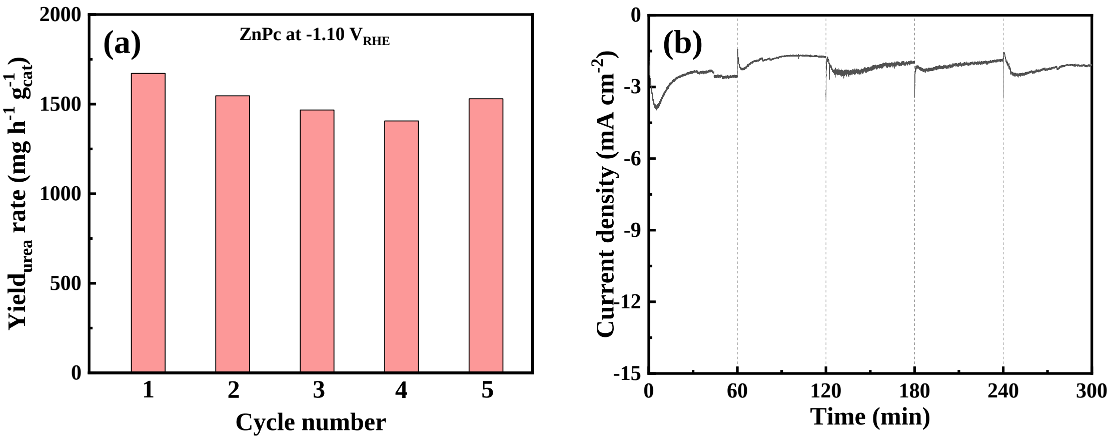

Figure S2. (a) Yield rates of urea for ZnPc at -1.10 V_{RHE} for continuous five cycles and (b) the corresponding current density *vs.* time curves.

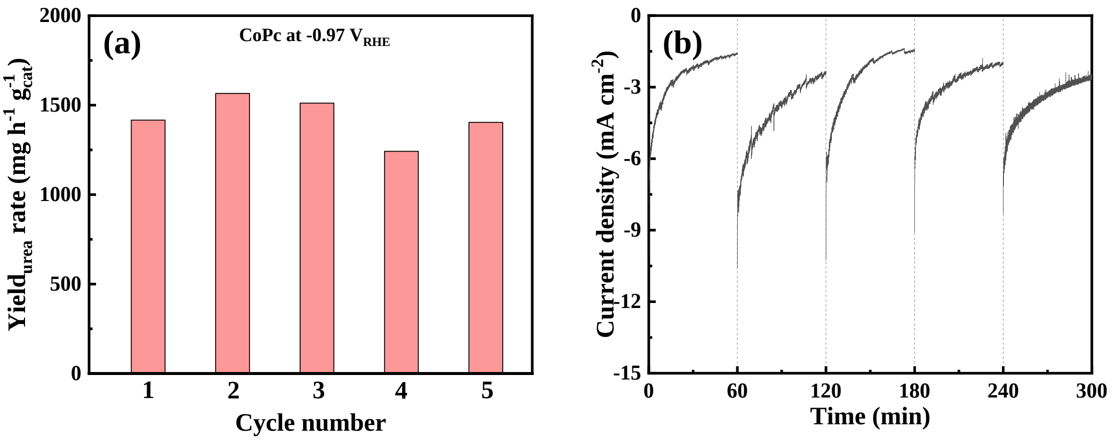

Figure S3. (a) Yield rates of urea for CoPc at -0.97 V_{RHE} for continuous five cycles and (b) the corresponding current density *vs.* time curves.

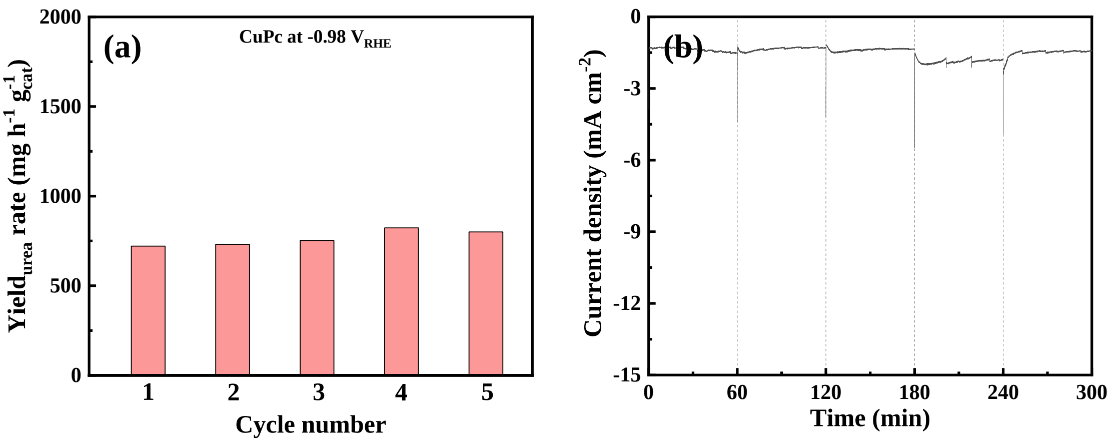

Figure S4. (a) Yield rates of urea for CuPc at -0.98 V_{RHE} for continuous five cycles and (b) the corresponding current density *vs.* time curves.

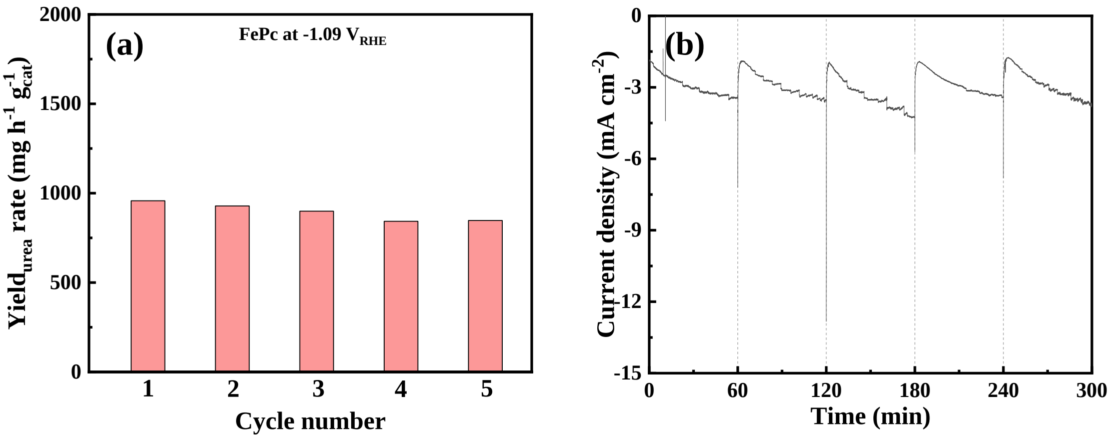

Figure S5. (a) Yield rates of urea for FePc at -1.09 V_{RHE} for continuous five cycles and (b) the corresponding current density *vs.* time curves.

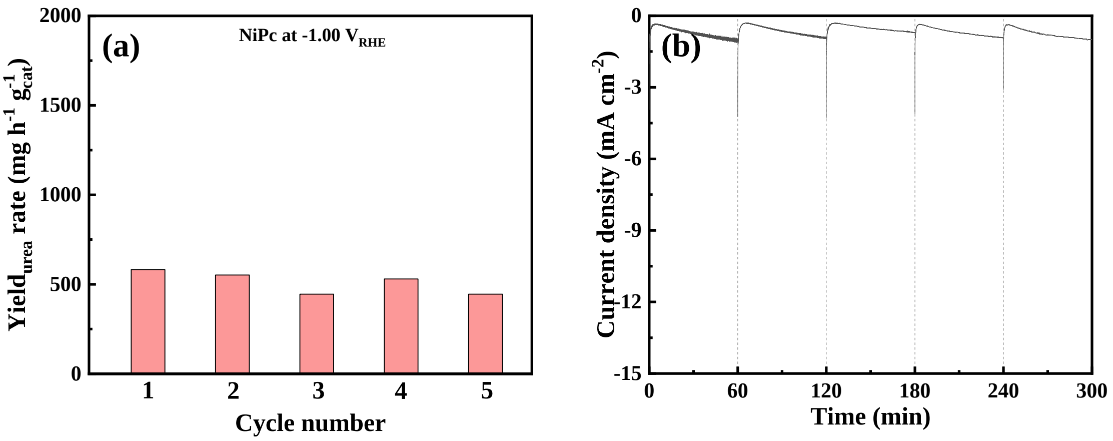

Figure S6. (a) Yield rates of urea for NiPc at -1.00 V_{RHE} for continuous five cycles and (b) the corresponding current density *vs.* time curves.

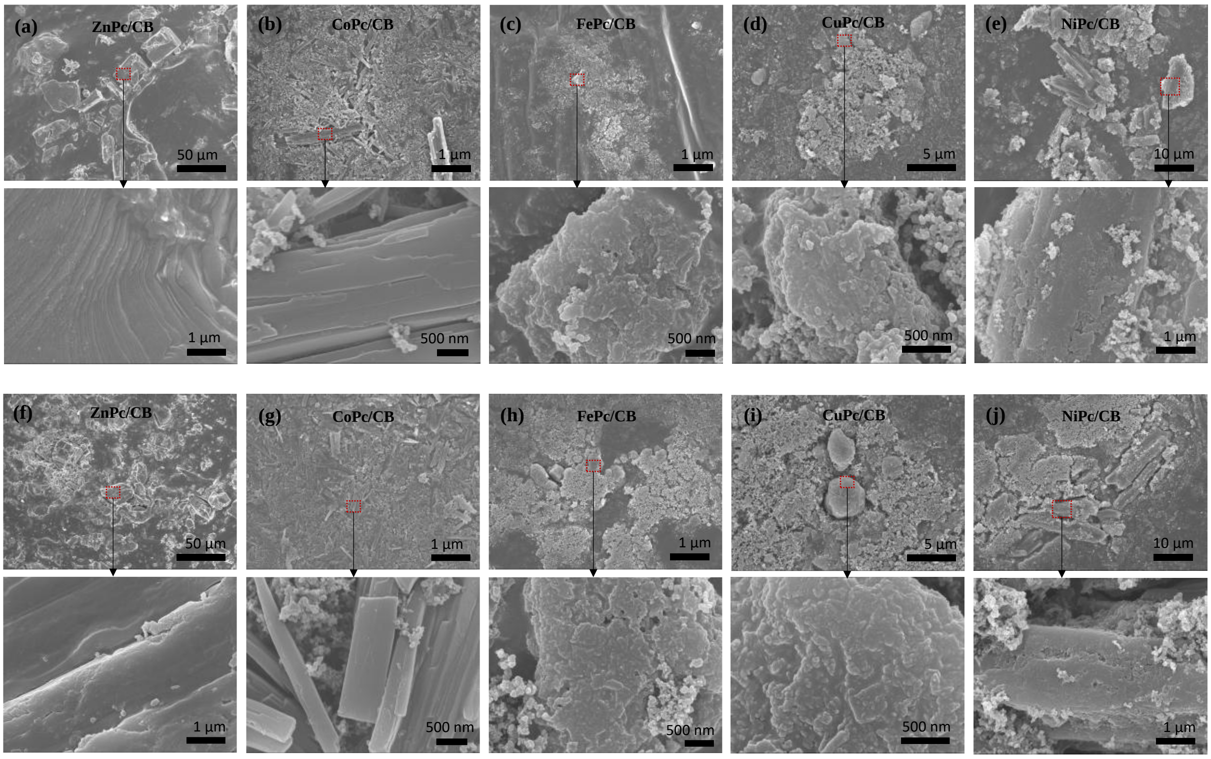

Figure S7. Scanning electron microscope (SEM) images of (a, f) ZnPc/carbon black (CB), (b, g) CoPc/CB, (c, h) FePc/CB, (d, i) CuPc/CB, and (e, j) NiPc/CB before (a-e) and after (f-j) stability tests.

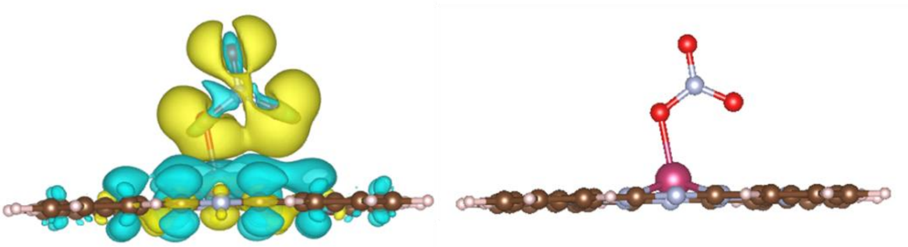

Figure S8. The Bader charge transfer of $*NO_3$ adsorbed on ZnPc with a net charge of 0.70e.

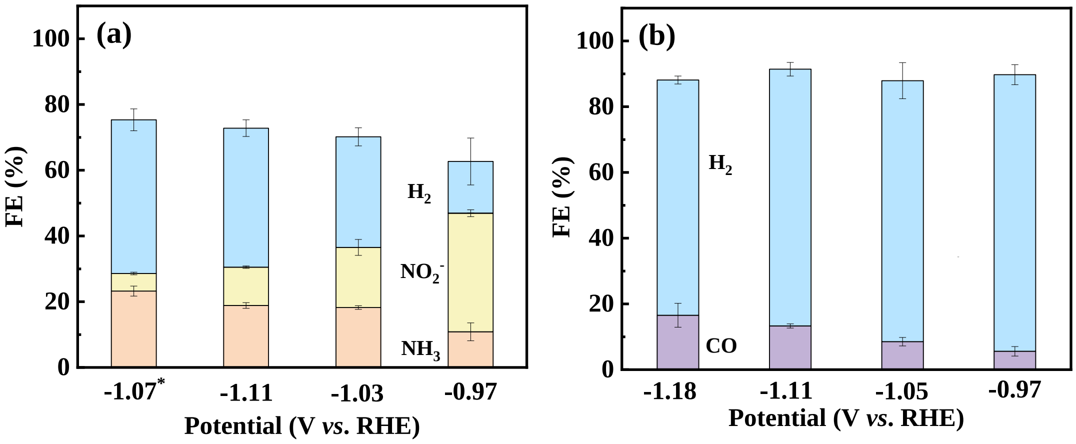

Figure S9. FEs of ZnPc at different potentials tested in (a) Ar-saturated 0.1M $KHCO_3$/0.01M KNO_3 and (b) CO_2-saturated 0.11M $KHCO_3$. (* The potential after iR compensation was calculated as the second negative one due to the large current density under this potential, though the applied potential was the most negative one.)

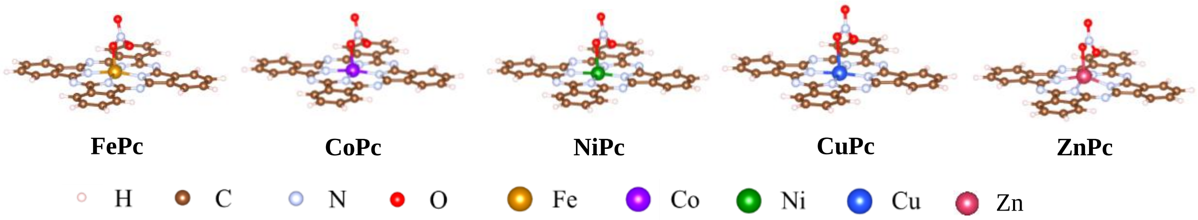

Figure S10. Configurations of the $*NO_3$ intermediate adsorbed on the MPc catalysts.

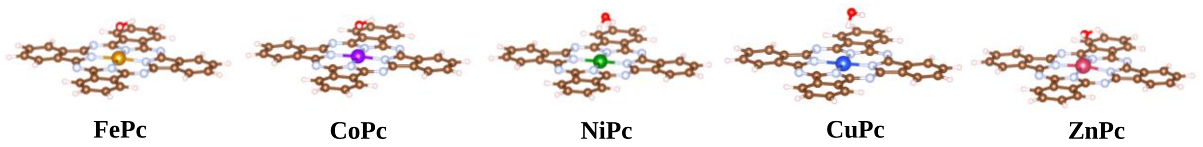

Figure S11. Configurations of the *H_2O adsorbed on the MPc catalysts. (The color notation of atoms keeps in consistence throughout this work.)

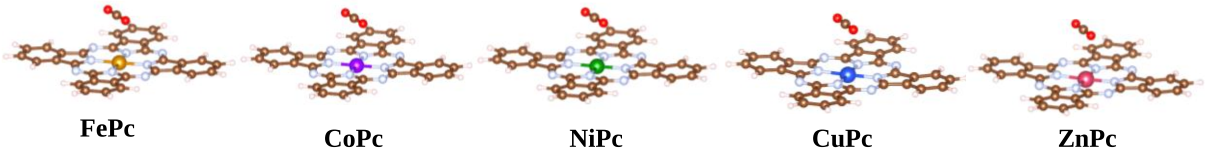

Figure S12. Configurations of the *CO_2 adsorbed on the MPc catalysts.

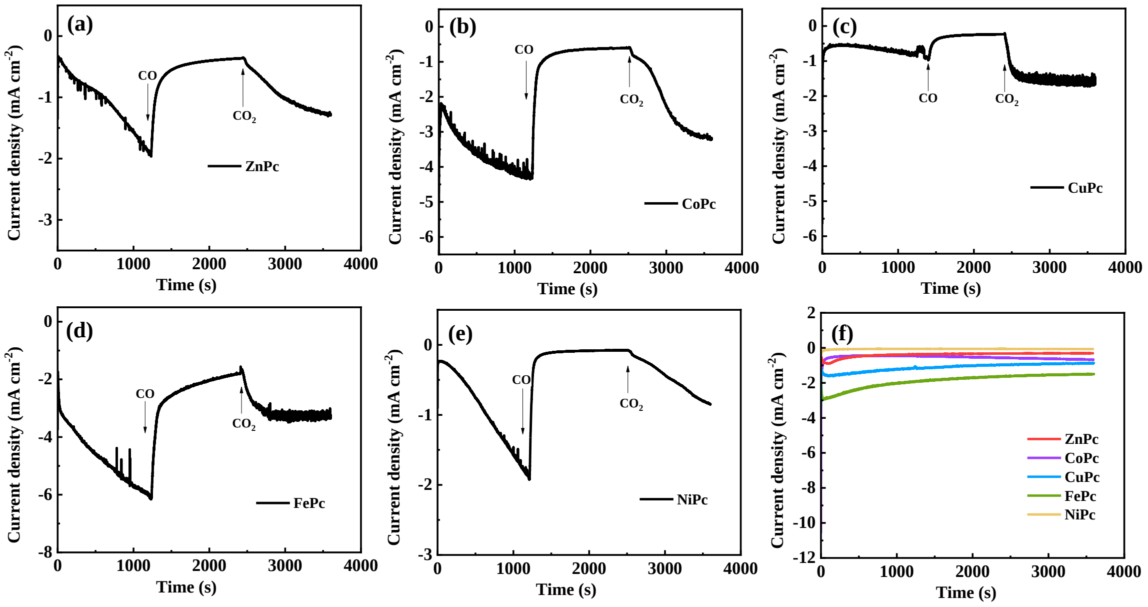

Figure S13. The current density *vs.* time curves of different MPc catalysts at an applied potential of -1.0 V_{RHE} without iR compensation with CO and CO_2 shifting: (a) ZnPc, (b) CoPc, (c) CuPc, (d) FePc, and (e) NiPc; (f) the current density *vs.* time curves of different MPc catalysts under the gas flow of CO for 1 h.

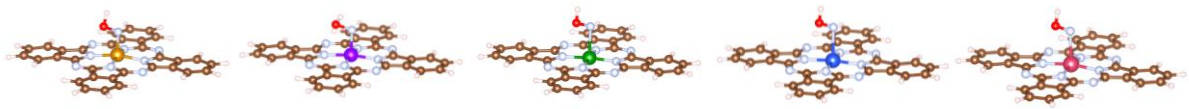

FePc **CoPc** **NiPc** **CuPc** **ZnPc**

Figure S14. Configurations of the *NOH intermediate adsorbed on the MPc catalysts.

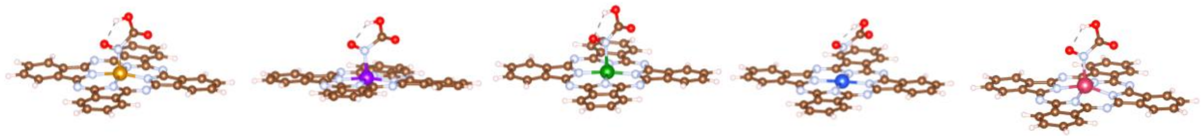

FePc **CoPc** **NiPc** **CuPc** **ZnPc**

Figure S15. Configurations of the *NOCOOH intermediate adsorbed on the MPc catalysts.

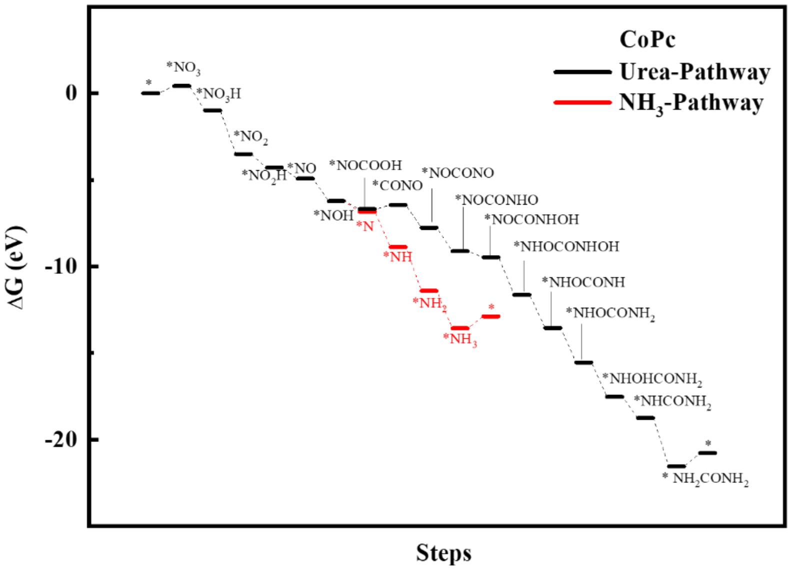

Figure S16. The Gibbs free energy change of the full urea pathway and that of NO_3RR pathway ending at the *N step on CoPc catalyst (pH = 6.8, U = -1.0 V_{RHE}).

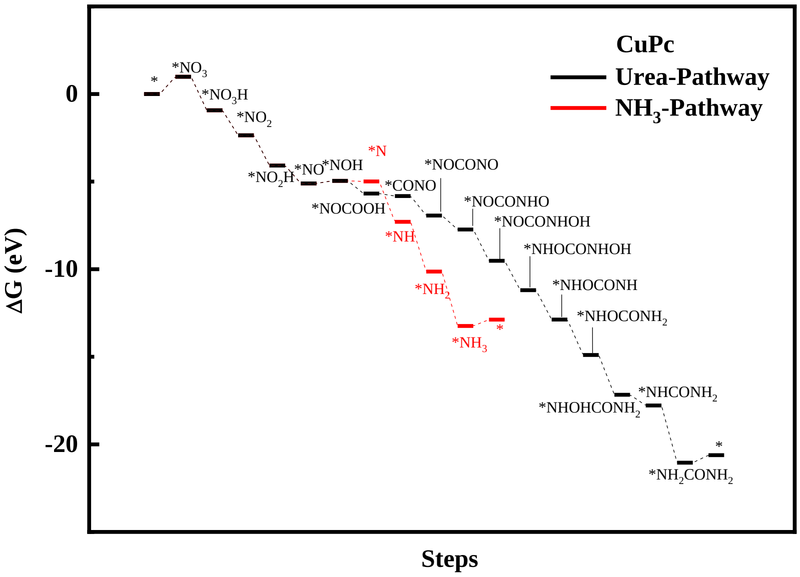

Figure S17. The Gibbs free energy change of the full urea pathway and that of NO3RR pathway ending at the *N step on CuPc catalyst (pH = 6.8, U = -1.0 V_{RHE}).

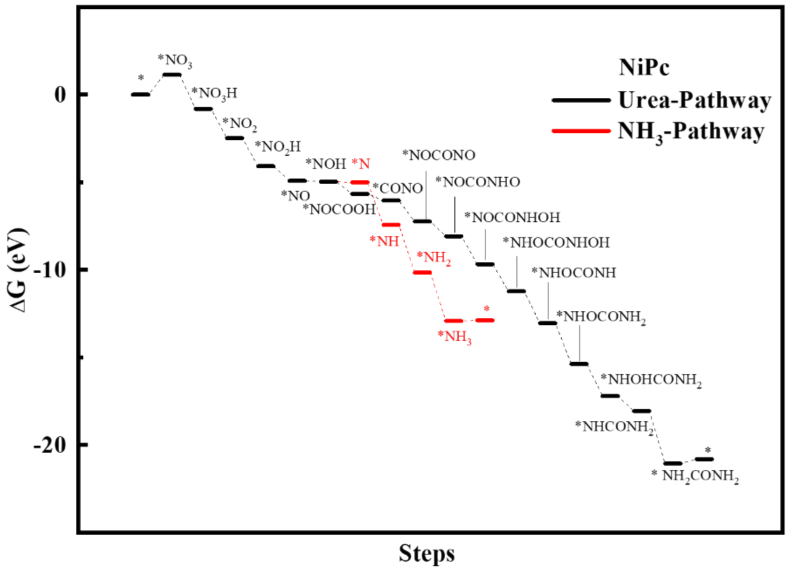

Figure S18. The Gibbs free energy change of the full urea pathway and that of NO3RR pathway ending at the *N step on NiPc catalyst (pH = 6.8, U = -1.0 V_{RHE}).

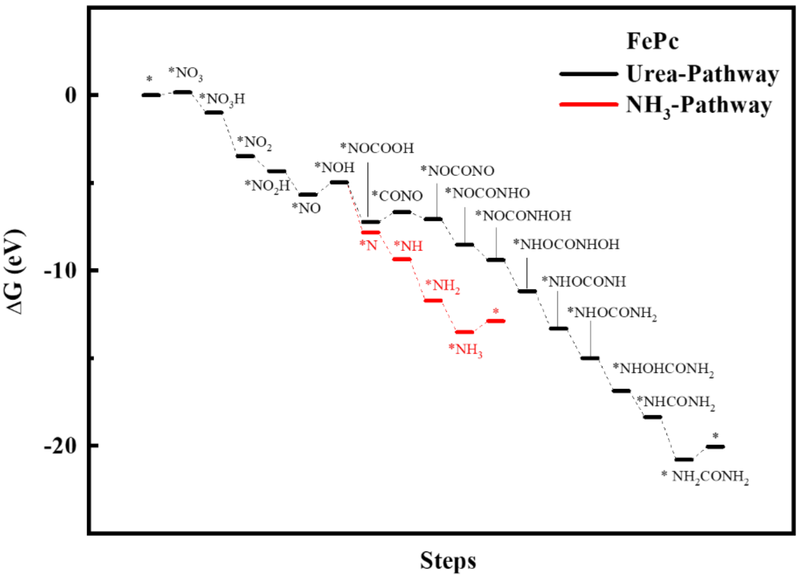

Figure S19. The Gibbs free energy change of the full urea pathway and that of NO_3RR pathway ending at the *N step on FePc catalyst (pH = 6.8, U = -1.0 V_{RHE}).

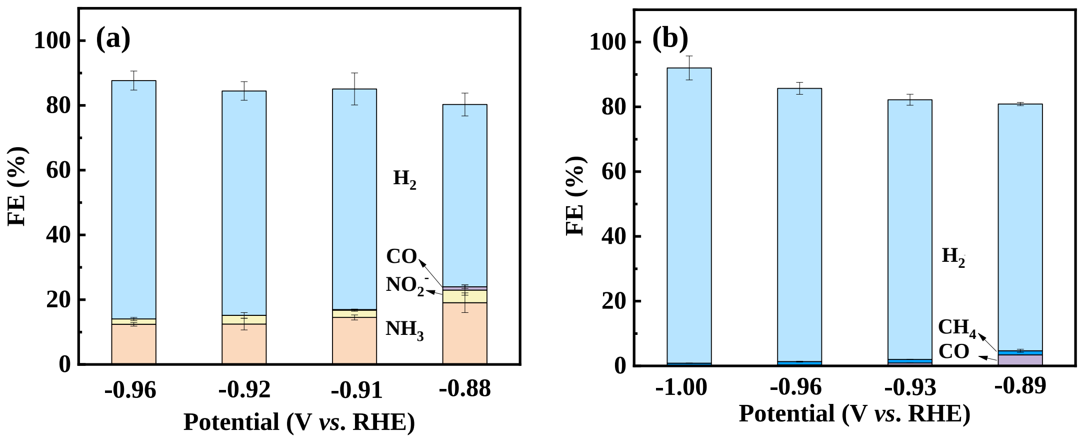

Figure S20. FEs of CoPc at different potentials tested in (a) Ar-saturated 0.1M $KHCO_3$/0.01M KNO_3 and (b) CO_2-saturated 0.11M $KHCO_3$.

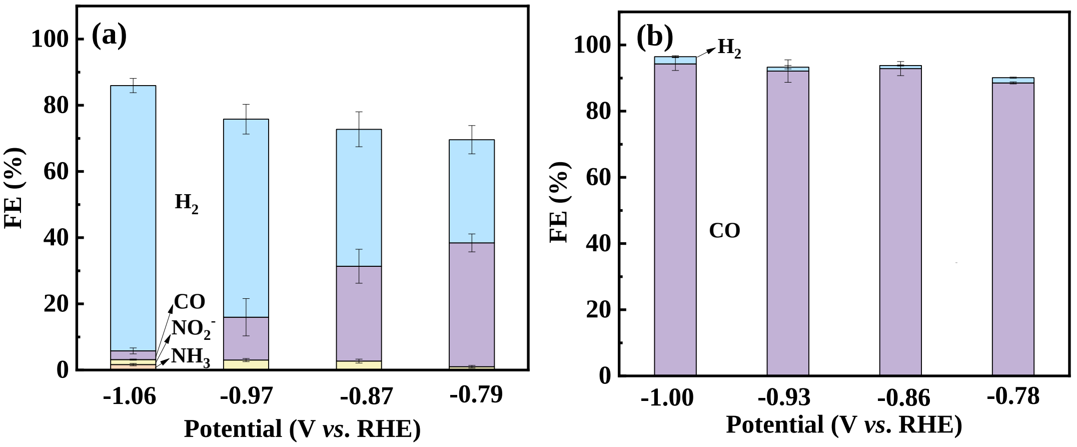

Figure S21. FEs of CuPc at different potentials tested in (a) Ar-saturated 0.1M $KHCO_3$/0.01M KNO_3 and (b) CO_2-saturated 0.11M $KHCO_3$.

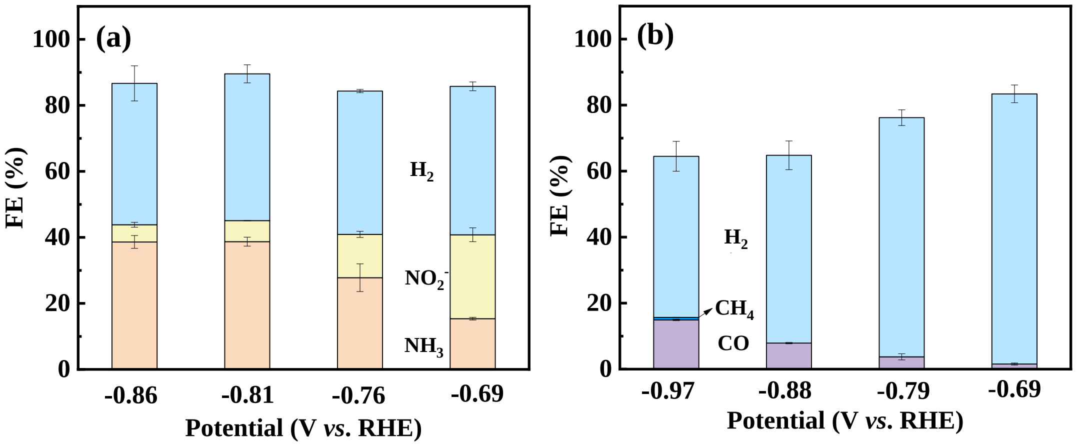

Figure S22. FEs of NiPc at different potentials tested in (a) Ar-saturated 0.1M $KHCO_3$/0.01M KNO_3 and (b) CO_2-saturated 0.11M $KHCO_3$.

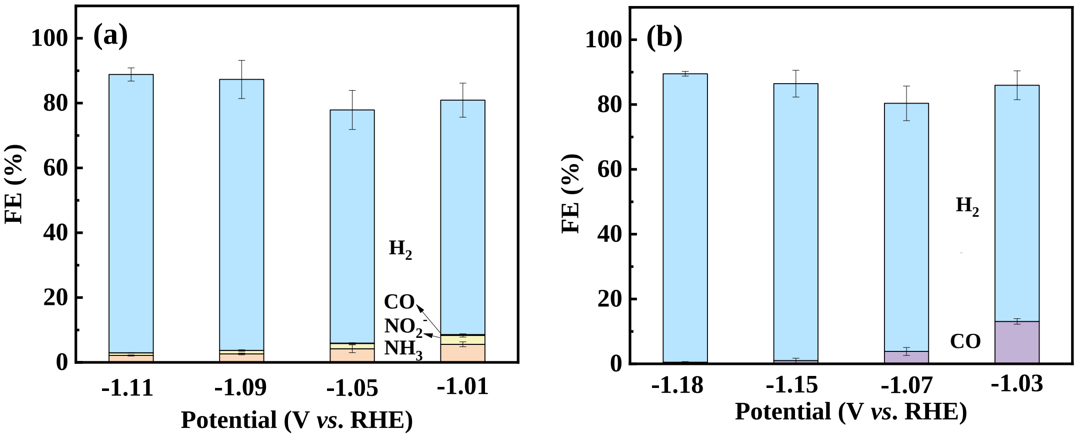

Figure S23. FEs of FePc at different potentials tested in (a) Ar-saturated 0.1M $KHCO_3$/0.01M KNO_3 and (b) CO_2-saturated 0.11M $KHCO_3$.

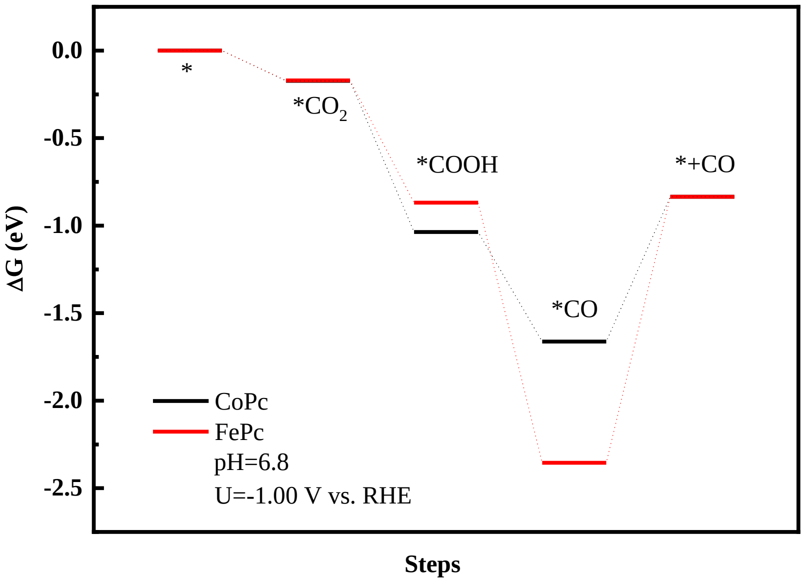

Figure S24. The Gibbs free energy change of CO_2RR process on CoPc and FePc.

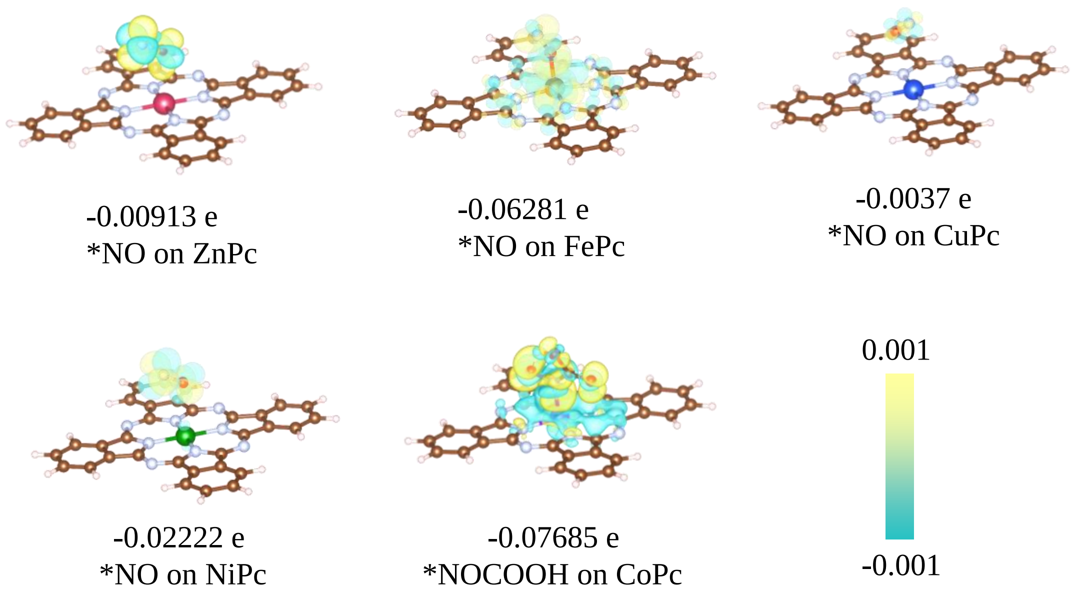

Figure S25. Charge density difference distributions of *NO on ZnPc, FePc, CuPc, and NiPc, and *NOCOOH on CoPc.

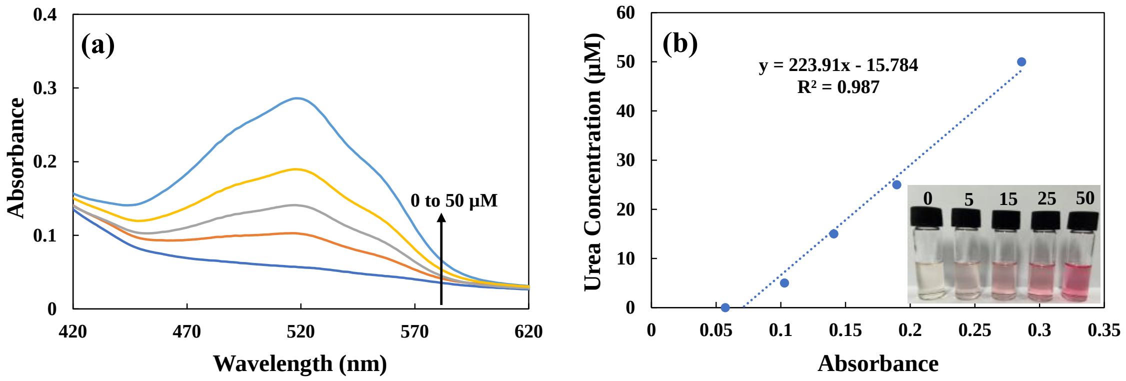

Figure S26. UV-vis absorption spectra cited from our previous work: (a) of standard samples in various urea concentrations and the corresponding typical calibration curves (b) with the inset of standard samples (μmol L^{-1}). Reproduced with permission.[1] Copyright 2025, John Wiley and Sons.

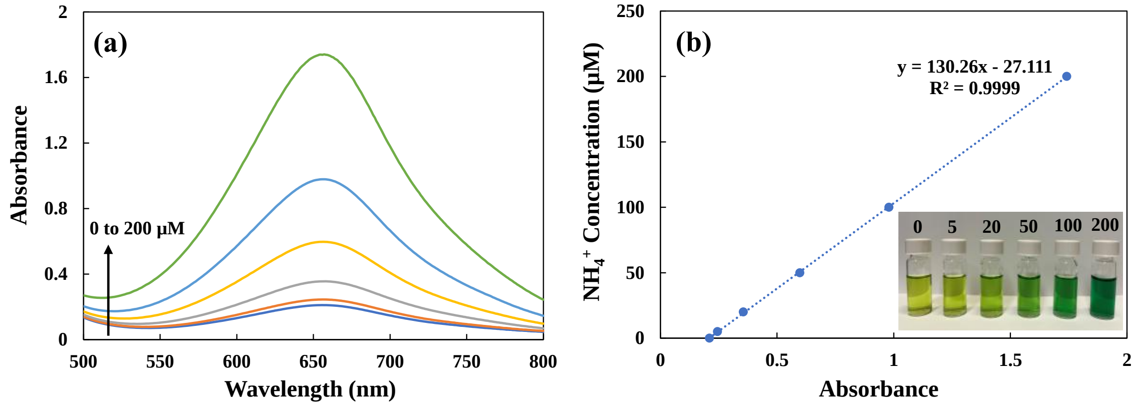

Figure S27. (a) UV-vis absorption spectra cited from our previous work of standard samples in various NH_4^+ concentrations and (b) the corresponding typical calibration curves with the inset of standard samples (μmol L^{-1}). Reproduced with permission.[1] Copyright 2025, John Wiley and Sons.

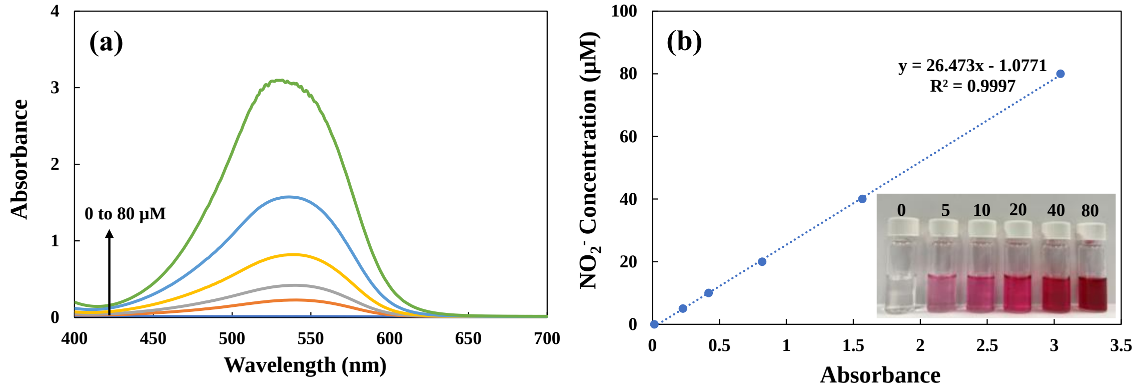

Figure S28. UV-vis absorption spectra cited from our previous work: (a) of standard samples in various NO_2^- concentrations and the corresponding typical calibration curves (b) with the inset of standard samples (μmol L^{-1}). Reproduced with permission.[1] Copyright 2025, John Wiley and Sons.

Table S1. The concentrations of corresponding metal ions in electrolytes for different MPc catalysts after 1 hour catalytic reaction measured by ICP-MS.

Sample	ZnPc	CoPc	FePc	CuPc	NiPc
Metal ion	Zn^{2+}	Co^{2+}	Fe^{3+}	Cu^{2+}	N^{2+}
Concentration (ppb)	0.50	0.85	1.02	0.24	0.97

Table S2. The adsorption energy of different species on active sites of different MPc catalysts.

MPc	Adsorption energy (eV)		
	$*NO_3^-$	$*CO_2$	$*H_2O$
ZnPc	0.69	-0.18	-0.41
NiPc	1.14	-0.20	-0.21
CoPc	0.43	-0.17	-0.46
CuPc	0.92	-0.31	-0.05
FePc	0.17	-0.17	-0.52

Table S3. The Gibbs energy change of different intermediates for MPcs in the calculated pathways toward urea production and NH_3 formation.

	Gibbs energy change (eV)				
	ZnPc	CoPc	CuPc	NiPc	FePc
*NO_3	0.69	0.43	0.98	1.14	0.17
*NO_3H	-0.90	-0.99	-0.93	-0.81	-1.00
*NO_2	-2.19	-3.52	-2.36	-2.48	-3.49
*NO_2H	-4.17	-4.30	-4.09	-4.09	-4.33
*NO	-4.98	-4.93	-5.11	-4.91	-5.68
*NOH	-4.99	-6.22	-4.96	-4.97	-4.97
*NOCOOH	-5.84	-6.69	-5.68	-5.67	-7.24
*CONO	-6.12	-6.44	-5.83	-6.04	-6.67
*NOCONO	-7.04	-7.76	-6.94	-7.24	-7.08
*NOCONHO	-7.85	-9.12	-7.74	-8.08	-8.53
*NOCONHOH	-9.75	-9.46	-9.52	-9.68	-9.40
*NHOCONHOH	-11.44	-11.64	-11.20	-11.23	-11.18
*NHOCONH	-13.46	-13.55	-12.88	-13.05	-13.32
*$NHOCONH_2$	-15.52	-15.54	-14.90	-15.38	-15.00
*$NHOHCONH_2$	-17.51	-17.53	-17.17	-17.21	-16.86
*$NHCONH_2$	-18.54	-18.75	-17.78	-18.05	-18.36
*NH_2CONH_2	-21.47	-21.54	-21.05	-21.05	-20.80
*N	-4.72	-6.85	-4.99	-5.00	-7.83
*NH	-7.33	-8.87	-7.30	-7.43	-9.37
*NH_2	-10.38	-11.41	-10.14	-10.16	-11.72
*NH_3	-13.48	-13.57	-13.24	-12.92	-13.52

Table S4. The adsorption energy of different species for forming the descriptor $\Delta G_{*HOOCNO}$-ΔG_{*N}-ΔG_{*COOH}+ΔG_{*H2O}.

MPc	Adsorption energy (eV)			
	*HOOCNO	*N	*COOH	$*H_2O$
ZnPc	-0.85	0.28	1.02	-0.41
NiPc	-0.70	-0.03	0.85	-0.21
CoPc	-0.47	-0.62	-0.86	-0.46
CuPc	-0.72	-0.03	0.97	-0.05
FePc	-2.27	-2.86	-0.70	-0.52

References

[1] Q. Zhao, X. Lu, Y. Wang, S. Zhu, Y. Liu, F. Xiao, S. X. Dou, W.-H. Lai M. Shao, *Sustainable and high-rate electrosynthesis of nitrogen fertilizer*, *Angew. Chem. Int. Ed.* **2023**, 62, e202307123.
